# Differential Associations Between Distinct Components of Cognitive Function and Mobility: Implications for Understanding Aging, Turning and Dual-Task Walking

**DOI:** 10.3389/fnagi.2019.00166

**Published:** 2019-07-02

**Authors:** Preeti Sunderaraman, Inbal Maidan, Tal Kozlovski, Zoltan Apa, Anat Mirelman, Jeffrey M. Hausdorff, Yaakov Stern

**Affiliations:** ^1^Cognitive Neuroscience Division, Gertrude H. Sergievsky Center, Taub Institute for Research on Alzheimer’s Disease and the Aging Brain, Columbia University Irving Medical Center, New York, NY, United States; ^2^Center for the Study of Movement Cognition and Mobility, Neurological Institute, Tel Aviv Sourasky Medical Center, Tel Aviv, Israel; ^3^Department of Neurology and Neurosurgery, Sackler Faculty of Medicine, and Sagol School of Neuroscience, Tel Aviv University, Tel Aviv, Israel; ^4^Department of Physical Therapy, Sackler Faculty of Medicine, and Sagol School of Neuroscience, Tel Aviv University, Tel Aviv, Israel; ^5^Rush Alzheimer’s Disease Center and Department of Orthopedic Surgery, Rush University Medical Center, Chicago, IL, United States; ^6^Department of Neurology, Columbia University Irving Medical Center, New York, NY, United States

**Keywords:** mobility, gait, cognition, aging, elderly, executive function, processing speed, vocabulary

## Abstract

**Objective:**

Cognition and mobility are interrelated. However, this association can be impacted by the specific facets of cognition and mobility that are measured, and further by the different task conditions, e.g., single- versus dual-task walking, under which these associations are evaluated. Systematically studying the multiple facets of cognitive-mobility associations under both the task conditions is critical because both cognition and mobility change with age and pose significant risks associated with falls, morbidity, and disability.

**Methods:**

Using a cross-sectional, prospective study design, data from 124 healthy adults [mean age (SD) = 61.51 (11.90); mean education (SD) = 15.94 (2.18)] were collected. A comprehensive battery of cognitive tests was administered, and gait was assessed using a small, lightweight, three-axis accelerometer with a gyroscope.

**Analytical Plan:**

Data were transformed, and only relatively strong relationships survived after strict statistical criteria adjusting for multiple comparisons were applied. Spearman rho correlation coefficients were used to examine the matrix of correlations between the cognitive-motor variables while adjusting for age and gender.

**Results:**

Executive functions, processing speed, and language were associated with distinct facets of variability, pace, and asymmetry, especially under the dual-task walking condition. Both turns and transitions were also associated with cognition during the Timed Up and Go Task.

**Conclusion:**

Our results extend converging evidence of the involvement of executive functions and processing speed in specific aspects of mobility, along with the role of language. The study has important implications for aging in terms of both assessment and rehabilitation of cognition and gait as well as for the emerging dual-tasking theories and the role of the neural pathways involved in mobility.

## Introduction

Cognition and mobility are both multifaceted. Whereas cognitive functions can be classified into executive function, memory, attention, language, and processing speed (Lezak et al., 2004), gait – a predominant aspect of mobility – can be subdivided into pace, rhythm, asymmetry, and variability. Transitions and turns reflect additional, independent and putatively distinct mobility domains. There is compelling evidence that these cognitive functions and mobility domains are interrelated, especially among older adults, however, systematic study of their inter-relationships is lacking.

Key components of mobility such as gait, transitions (e.g., sit to stand, stand to sit), and turns have all been related to cognition. For example, older adults with better executive function walked with increased gait speed (Yogev-Seligmann et al., 2008; Morris et al., 2016), and those with slower processing speed had reduced rhythm (Martin et al., 2012). Both executive function and processing speed are susceptible to age-related changes (Brickman et al., 2007; Glisky, 2007; Salthouse, 2010), while decrements in gait integrity (such as stability) are associated with increasing age (Terrier and Reynard, 2015). Moreover, prospective studies have shown that among older adults, changes in certain aspects of gait predict cognitive decline and dementia, while the converse has also been observed with executive function and memory (i.e., mostly verbal memory) predicting a decline in gait (Yogev-Seligmann et al., 2008; Montero-Odasso et al., 2012; Morris et al., 2016). Nonetheless, several studies have suggested that the exact nature of this cognitive-mobility relationship still needs to be fully elucidated (Morris et al., 2016; Montero-Odasso et al., 2018). One such approach is to examine these associations in a relatively large cohort of healthy individuals consisting of diverse age-ranges. If the findings of cognitive-mobility associations across diverse age-ranges parallel those previously found in older adults, clinicians, and researchers can then better understand the mechanisms associated with gait, especially given that early changes in cognition and gait have the potential to identify those at risk for falls and cognitive decline (Montero-Odasso et al., 2012).

The cognitive-mobility association can be influenced by the task condition, e.g., simple walking without any interference (single task condition) or walking while performing another task such as verbal fluency (dual-task condition) (Yogev-Seligmann et al., 2008). Dual-tasking is important to study for several reasons. Mechanistically, it can be used to probe the automaticity of gait. Clinically, it can be used to unmask subclinical changes before an actual diagnosis and, predict critical outcomes such as falls and disability (Beauchet et al., 2009; Verghese et al., 2012; Li et al., 2018). A systematic study of the relationship between cognitive function and mobility while taking into account the multi-faceted nature of these functions, with and without dual-tasking, is lacking.

In contrast to the many studies that have reported on the decline in gait speed during dual-tasking (Beauchet et al., 2009; Verghese et al., 2012), there is limited evidence describing the specific one-to-one relationships that link cognition and mobility taking into account both single and dual-tasking. Pace and the stride-to-stride variability of gait, along with acceleration measures of transitions and turns have been related to attention and processing speed (Maki and McIlroy, 2007; Lord et al., 2013; Mirelman et al., 2014). Deficits in executive function and processing speed were associated with increased falls in community-dwelling older adults (Herman et al., 2010), and those with clinical impairments such as Parkinson’s disease (Mirelman et al., 2013). In contrast, others reported that rhythm was related to memory (i.e., verbal memory) and that pace was more restricted to executive function, memory, and language (Holtzer et al., 2012). A recent review indicated that asymmetry was not associated with any cognitive domain and that rhythm was linked to processing speed, but consistent associations were not found between gait variability and cognition (Morris et al., 2016), despite previous work which suggests otherwise. Such diverse findings are obfuscated by the nature of the gait variables included in a specific study (e.g., emphasis on the pace, i.e., gait speed, domain but not others), the types of cognitive domains studied (i.e., inconsistencies in the cognitive battery used across studies), and the nature of the dual-task selected for study. In addition, the cognitive-mobility relationships varied with respect to the specific cohort in question. For example, one study found that pace was compromised to a higher extent in patients with non-amnestic MCI having executive function, attention and language deficits as compared to amnestic MCI patients (Verghese et al., 2008), whereas other studies found contrasting associations between pace and executive function among healthy older adults (Morris et al., 2016).

There is, therefore, a pressing need to systematically and simultaneously examine multiple facets of both cognition and mobility under different task conditions, i.e., single and dual-task walking using a spectrum of tests to measure multiple facets of cognition. The current study sought to parse out the differential associations in a healthy cohort by generating a relatively large matrix of the cognitive and motor measurements. Such an approach can improve the sensitivity to detect relationships between mobility and cognition.

## Materials and Methods

### Study Design and Participants

Data were collected prospectively from community-dwelling participants who were recruited through random market mailings. Following established procedures, participants were required to be native English speakers, strongly right-handed, and have a minimum of fourth-grade reading level (Stern et al., 2014). They were also screened for MRI contraindications, and hearing and visual impairment that would interfere with testing. Older participants were assessed for dementia or MCI using the Dementia Rating Scale (DRS). The Internal Review Board of the College of Physicians and Surgeons of Columbia University approved this study. Prior to the testing session, written informed consent was obtained from all participants and compensation was provided at the end of the study.

### Cognitive Measures

Four domains of cognition, i.e., executive function, memory, language, and processing speed, were measured using paper-and-pencil tests, and computerized tasks from the Reference Ability Neural Network Study (Stern et al., 2014). These domains were selected based on the results of latent constructs identified in large-scale studies of cognition across the adult lifespan (Salthouse, 2009). Table 1 displays the specific cognitive measures in each of the four domains. Further details about the tests can be found elsewhere (Kramer et al., 2014; Stern et al., 2014; Gazes et al., 2016; Habeck et al., 2016).

**TABLE 1:** Summary of cognitive measures under different task conditions.

	**Paper-and-pencil**	**NIH examiner battery**	**Salthouse and colleagues**
Executive function	Phonemic fluency Trial making test (Part B) WAIS III – Matrix reasoning WAIS III – Block design Wisconsin card sorting Test	Continuous performance test Set shifting test Flanker test Anti-saccade test	Paper folding Letter set Matrix reasoning
Processing speed	Grooved pegboard WAIS-R digit symbol		Digit symbol Letter comparison Pattern comparison Trial making test (Part A)
Memory	Selective reminding test		Logical memory Paired associates Word order
Language	AMNART WTAR WAIS-R vocabulary Animal fluency		Synonyms Antonyms Picture naming

### Mobility Measures

A small, lightweight, three-axis accelerometer with a gyroscope (DynaPort; McRoberts, The Hague, Netherlands) was worn on the lower back to quantify gait and the TUG test. The gait assessment included walking along a 20-m-long corridor for 1 min under two conditions: (1) preferred, usual-walking speed, and (2) dual-tasking, reciting words that start with the letter A while walking. The mean stride time, gait speed, stride length, stride and step time variability, stride regularity, step regularity, and step symmetry were quantified as previously described (Mirelman et al., 2013; Herman et al., 2014). The TUG test consisted of standing up from a chair, walking 3 m to a designated location at a normal pace, turning around, walking back, and sitting back down on the same chair. Acceleration signals were derived from three axes: vertical, mediolateral, and anterior–posterior. Angular velocities were derived from the gyroscope as yaw (rotation around the vertical axis), pitch (rotation around the mediolateral axis), and roll (rotation around the anterior–posterior axis) to derive quantitative measures for four subtasks: sit-to-stand and stand-to-sit transitions, walking, and turning (Weiss et al., 2011; Mirelman et al., 2014). Mobility measures were classified into six domains, transitions and turn domains from the TUG test, and variability, asymmetry, rhythm, and pace domains from the gait assessments (Lord et al., 2012).

### Statistical Analyses

Non-linear transformations were applied using a semi-automated R shiny application (Shachar et al., 2018). For the list of the full transformations applied to the cognitive and mobility variables, see Supplementary Table 1. In cases where an order reversing transformation was used, the transformed values were multiplied by −1. Hereafter, outliers were detected while adjusting for age and gender. The outliers were determined according to Bonferroni *p*-values for Studentized residuals (based on a *t*-test) that are smaller than 4/n (Weisberg, 2014). For the list of outliers removed, see Supplementary Table 2. In addition, to circumvent the problem of a limited range of values, we excluded variables on which participants obtained similar values with 80% frequency.

Spearman rho correlation coefficients were used to examine the correlations between the cognitive-motor variables while adjusting for age and gender. Specifically, the correlation between each motor variable and each grouping of cognitive variables by the same domain (e.g., executive function, language, speed, and memory) were determined as a group of hypotheses. Therefore, the number of hypothesis groups was equal to the number of motor variables multiplied by the number of cognitive domains, and the number of correlations within each hypothesis group was equal to the number of cognitive variables belonging to the same domain. The statistical analysis was computed in two stages. First, motor variables and cognitive domains were selected if the Simes *p*-value (Simes, 1986) calculated on all Spearman correlations belonged to the same cognitive and motor domains (hypothesis group), was smaller than 0.05. Second, *p*-values belonging to a chosen motor variable-cognitive domain group of hypotheses were tested with adjustment to control the false discovery rate (FDR), using the Benjamini-Hochberg (BH) method, at a level of 0.05 (Benjamini and Hochberg, 1995). This was performed for each of the chosen hypothesis groups separately. All statistical analyses were done using R software version 3.4.3.

## Results

### Participant Characteristics

**FIGURE 1:**
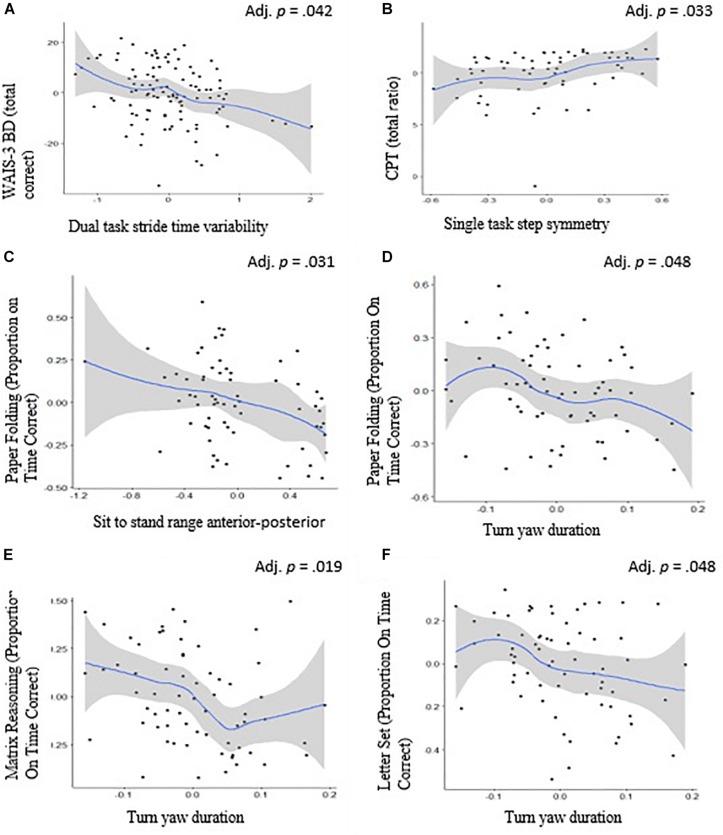
Scatterplots and the 95% confidence intervals (in gray) of Spearman’s rank correlation between executive functions and mobility. Relationship between **(A)** WAIS-3 BD (total correct) and dual task walk time stride variability, **(B)** CPT (total ratio) and Single task walk step symmetry, **(C)** Paper folding (proportion on time correct) and sit to stand range anterior–posterior, **(D)** Paper Folding (proportion on time correct) and turn yaw duration, **(E)** matrix reasoning (proportion on time correct) and turn yaw duration, and **(F)** letter set and turn yaw duration. Adjusted values reflect correction for multiple comparisons.

**FIGURE 2:**
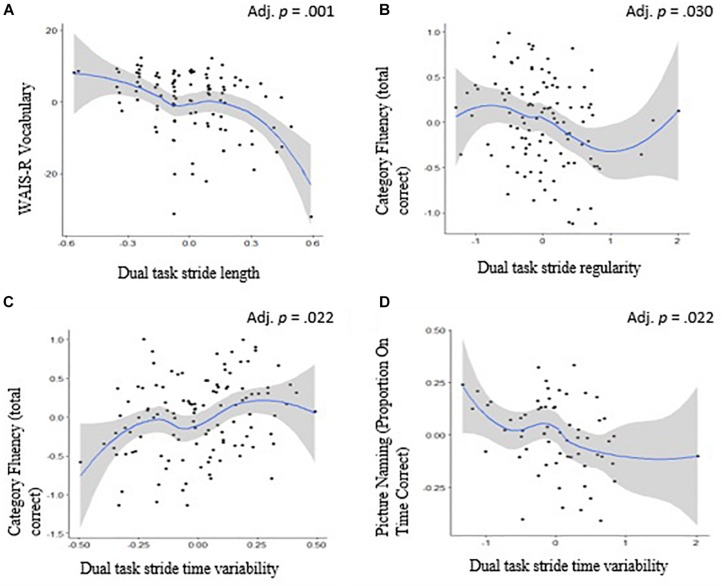
Scafterplots and the 95% confidence intervals (in gray) of Spearman’s rank correlation between language and mobility. Relationship between **(A)** WAIS-R vocabulary and dual task walk stride length, **(B)** category fluency (total correct) and dual task walk stride regularity, **(C)** category fluency (total correct) and dual task walk stride time variability, and **(D)** picture naming (proportion on time correct) and dual task walk stride time variability. Adjusted *p-*values reflect correction for multiple comparisons.

**FIGURE 3:**
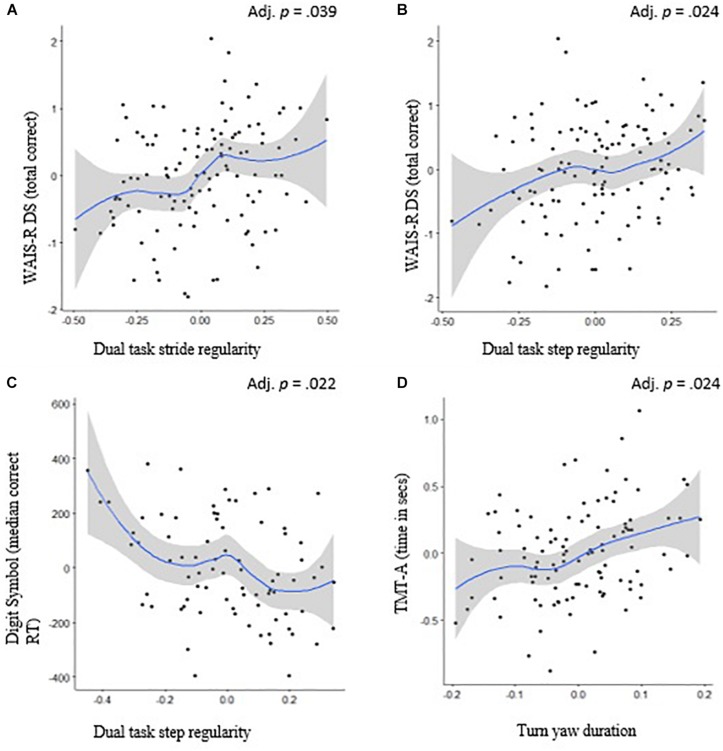
Scatterplots and the 95% confidence intervals (in gray) of Spearman’s rank correlation between processing speed and mobility. Relationship between **(A)** WAIS-R DS (total correct) and dual task walk stride regularity, **(B)** WAIS-R DS (total correct) and dual task walk step regularity, **(C)** Digit symbol (median correct RT) and dual task walk step regularity, and **(D)** TMT-A (time in seconds) and turn yaw duration. Adjusted *p-*values reflect correction for multiple comparisons.

One hundred and twenty-four, healthy adults were recruited from the community as a part of a large, ongoing study. Participants’ characterized are summarized in Table 2. Gait speed was an average of 0.93 (SD = 0.2) m/s. To illustrate the range of scores in our cohort, the scatterplots of the cognitive-mobility variables that were significantly correlated with each other are presented in Figures 1–3.

**TABLE 2:** Summary of demographic characteristics (*N* = 124).

	**Mean (SD), range**
Age (years)	61.51 (11.90), 27–80
Education (years)	15.94 (2.18), 11–20
Gender, Females (*n*, %)	70 (56.5%)
**Race (*n*, %)**	
Caucasian	90 (72.6%)
Black/African American	29 (23.4%)
Asian	1 (0.8%)
Other	4 (3.2%)
Dementia Rating Scale	140.24 (3.23)

### Associations of Cognition With Mobility

Tables 3, 4 provide information on the mean (SD) and ranges for the mobility and cognitive measures, respectively. Table 5 summarizes the correlation results of the cognitive-motor relationships. Briefly, executive function was inversely correlated with turn duration (*p* = 0.048), range of sit-to-stand (*p* = 0.031), dual-task stride time variability (*p* = 0.042), and dual-task step asymmetry (*p* = 0.033). Language was correlated with dual-task stride time variability (*p* = 0.022), dual-task stride regularity (*p* = 0.030), and most strongly with dual-tasking pace (i.e., stride length) (*p* = 0.001). Processing speed was associated with dual-task step regularity and dual-task stride regularity (*p* < 0.05) and turn duration (*p* = 0.024). Memory was not significantly related to any of the mobility measures (*p* > 0.06).

**TABLE 3:** Means, SDs, ranges for the motor measures.

		**Mean**	**SD**	**Range**
**Variability**				
	Single task stride regularity (NU)	0.75	0.1	0.28–0.92
	Single task stride time variability (%)	1.6	0.73	0.46–4.08
	Dual task stride regularity (NU)	0.6	0.19	0.09–0.92
	Dual task stride time variability (%)	2.73	2.39	0.45–16.43
**Transition**				
	Sit to stand range anterior–posterior (g)	1.78	1.8	16–8.53
	Sit to stand jerk anterior–posterior (g/s)	2.47	5.02	−2:55 to 21:74
	Sit to stand pitch amplitude (°/s)	−61:87	38.37	−158:39 to 94:24
	Stand to sit pitch amplitude (°/s)	58.3	28.38	−42:14 to 133:93
**Turn**				
	Turn yaw amplitude (°/s)	182.2	37.8	0.08–227.32
	Turn yaw duration (s)	1.63	0.44	0.74–3.40
**Asymmetry**				
	Single task step regularity (NU)	0.59	0.15	0.23–0.87
	Single task step symmetry (NU)	0.77	0.23	0–1.39
	Dual task step symmetry (NU)	0.87	0.35	0.02–2.25
	Dual task step regularity (NU)	0.52	0.18	0.06–0.86
**Rhythm**				
	Single task stride average (s)	1.12	0.1	0.86–1.41
	Dual task stride average (s)	1.23	0.16	0.88–1.73
**Pace**				
	Single task stride length (m)	1.13	0.2	0.84–1.83
	Single task gait speed (m/s)	0.93	0.2	0.66–1.71
	Dual task stride length (m)	1.15	0.2	0.75–2.00
	Time up go anterior posterior duration (s)	10.8	2.11	6.27–18.27
	Time up go duration (s)	6.15	1.85	2.38–14.84

*NU, no units; g, gravitational force per unit mass.*

**TABLE 4:** The means, SDs and ranges for the cognitive measures.

	**Executive function measures**	**Mean**	**SD**	**Range**	**Speed measures**	**Mean**	**SD**	**Range**	**Memory measures**	**Mean**	**SD**	**Range**	**Language measures**	**Mean**	**SD**	**Range**
Paper-and-pencil	Phonemic fluency (total correct)	47.04	12.85	19–77	GP-D, total time (in seconds)	82.73	27.41	50–198	SRT Last Trial (total correct)	9.87	2.15	0–12	AMNART errors	10.25	9.05	0–41
	TMT-B (time in seconds)	70.23	33.92	28–300	GP-NP, total time (in seconds)	93.04	29.37	56–213	SRT delayed recall	8.27	2.85	0–12	WTAR (total correct)	40.52	9.35	10–50
	WAIS-3 MR (total correct)	15.79	5.25	4–26	TMT-A (time in seconds)	27.72	10.69	9–79					WAIS-R vocabulary	58.18	9.60	17–70
	WAIS-3 BD (total correct)	38.52	12.71	4–65	WAIS-R DS (total correct)	52.13	12.94	28–93					Category fluency (total correct)	22.70	5.27	13–37
	WCST perseverative errors (total score)	6.48	6.24	1–28												
RAAN battery	Paper folding (proportion on time correct)	0.51	0.26	0.05–1	Digit symbol (median correct RT)	1571.57	233.41	999.5–2150	Logical memory (proportion on time correct)	0.72	0.19	0.06–1	Synonyms (proportion on time correct)	0.69	0.20	0.25–1
	Letter set (proportion on time correct)	0.72	0.22	0.17–1	Letter comparison (median correct RT)	1731.30	238.33	1150–2384.5	Paired associates (proportion on time correct)	0.68	0.22	0.2–1	Antonyms (proportion on time correct)	0.58	0.23	0.07–0.93
	Matrix reasoning (proportion on time correct)	0.47	0.27	0.05–1	Pattern comparison (median correct RT)	1608.31	241.77	1154–2409	Word order (proportion on time correct)	0.45	0.19	0.05–0.95	Picture naming (proportion on time correct)	0.56	0.19	0.1–0.9
NIH examiner battery	CPT correct ratio	97.91	3.49	76–100												
	Set-shifting, shift trials (ms)	7.95	0.71	5.83–9.12												
	Flanker (adjusted composite score)	8.40	0.66	5.63–9.42												

*TMT-A, trial making test, Part A; TMT-B, trial making test, Part B; WAIS-3 MR, matrix reasoning test from WAIS III; WAIS-3 BD, block design test from WAIS III; WCST, Wisconsin card sorting test; GP-D, grooved pegboard, dominant hand; GP-ND, grooved pegboard, non-dominant hand; WAIS-R DS, digit symbol from WAIS-R; SRT, selective reminding test; CPT, continuous performance test.*

**TABLE 5:** Summary of the associations between motor-cognitive measures.

**Cognitive domain**		**Motor domain**		**Adjusted *p*-value**
**Executive function**				
	WAIS-3 BD (total correct)	Variability	Dual task stride time variability	0.042
	CPT (total ratio)	Asymmetry	Single task step symmetry	0.033
	Paper folding (proportion on time correct)	Transition	Sit to stand range anterior–posterior	0.031
	Paper folding (proportion on time correct)	Turn	Turn yaw duration	0.048
	Matrix reasoning (proportion on time correct)	Turn	Turn yaw duration	0.019
	Letter set (proportion on time correct)	Turn	Turn yaw duration	0.048
**Language**				
	WAIS-R vocabulary	Pace	Dual task stride length	0.001
	Category fluency (total correct)	Variability	Dual task stride regularity	0.030
	Category fluency (total correct)	Variability	Dual task stride time variability	0.022
	Picture naming (proportion on time correct)	Variability	Dual task stride time variability	0.022
**Processing speed**				
	WAIS-R DS (total correct)	Variability	Dual task stride regularity	0.039
	WAIS-R DS (total correct)	Asymmetry	Dual task step regularity	0.024
	Digit symbol (median correct RT)	Asymmetry	Dual task step regularity	0.022
	TMT-A (time in seconds)	Turn	Turn yaw duration	0.024

## Discussion

Using a relatively comprehensive set of cognitive-mobility measures and a rather conservative statistical approach, we observed two striking results. First, in line with previous studies, executive function and processing speed were predominantly related to gait variability and turns, while memory was not significantly related to any of the mobility domains (Hausdorff et al., 2005; Iersel et al., 2008; Martin et al., 2012). Secondly, language appears to play a relatively strong role across different gait variability measures, specifically during the dual task condition.

In contrast to the current findings, two previous studies from the same group of researchers found that memory was associated with mobility. Specifically, memory was related to decreased gait speed during both single- and dual-task conditions (Holtzer et al., 2006; Tripathi et al., 2019). Based on these previous studies, one may argue that the memory tests used in the current study were not adequately representative of the memory domain to detect associations (Stern et al., 2014). However, studies using other memory tests such as the Hopkins Verbal Learning Test, the Rey Complex Figure test, and the Paired Associates Learning test also did not find independent associations between gait and memory (Hausdorff et al., 2005; Iersel et al., 2008; Martin et al., 2012). Population characteristics may be a partial explanatory factor as both of the previous studies (Holtzer et al., 2006; Tripathi et al., 2019) included older adults with comorbid conditions (e.g., arthritis, hypertension, diabetes, and a few with neurological diagnosis such as MCI and stroke), while we focused on healthy subjects. Another possible explanation is that the cognitive demand associated with the task itself and the instructions given during the task may be intrinsically different and load differently on specific cognitive abilities. Specifically, explicit instructions to focus on both on walking and the task of reciting alternate letters of the alphabet used by Holtzer et al. (2006) and Tripathi et al.’s (2019) studies may require a higher level of episodic and working memory resources versus generating words that begin with a specific letter which may load more on executive function and working memory. However, this explanation is speculative and needs to be empirically evaluated. The common finding for the lack of association between memory and gait found across most studies is based on the idea that walking, by itself, is a complex activity requiring minimal aspects of memory functioning (Hausdorff et al., 2005). As such, walking relies on executive functioning and multitasking to a larger extent than other aspects of cognition. Indeed, the relationship of memory to gait is complex especially once a non-walking task (for the dual-task condition) is introduced.

For executive functions, differential relationships were found with mobility such that it was associated with dual-task condition stride time variability, single-task condition step symmetry, and turn duration and sit-to-stand transition during the TUG task. Dual-task walking and turns are recognized to be complex motor functions with relatively higher cognitive demands. Both walking during the dual-task condition and, transitioning and turning during the single-task condition can thus make walking less safe and increase the risk of falls (Yogev-Seligmann et al., 2008; Herman et al., 2010; Maidan et al., 2017). While turns were associated with several executive function tasks, only one executive function task was related to transition during a sit to stand task. Transitioning from a sitting to standing position requires a series of complex motor skills such as moving forward with the body while seated, accelerating in the vertical plane while pushing upward from the seat and moving the body upward, and then slowing the momentum to achieve stability to stand (Janssen et al., 2002; Mirelman et al., 2014). Comparatively, turning requires a series of other complex motor skills involving coordination of the limbs, aligning and stabilizing one’s posture with various aspects of gait, and manipulating specific subsets of movements are required. Additionally, turning also requires greater involvement of visual processing along with intact balance and spatial perception (Mirelman et al., 2014). Studies have found that while both transitions and turns are associated with executive functioning, turns are additionally associated with processing speed, and visuo-spatial perception (Herman et al., 2011; Mirelman et al., 2014; Mancini et al., 2016; Mellone et al., 2016). Overall, our findings on executive functions and mobility suggest that, in healthy individuals, relatively lowered cognitive performance may be linked to increased risk of gait alterations during the performance of these complex motor functions, or that lowered cognition may represent a higher vulnerability to gait disturbances. While cause and effect cannot be evaluated in this cross-sectional analyses, the present findings, consistent with previous studies (Brustio et al., 2017), highlight the relationship between executive functions and specific aspects of mobility.

Regarding language, category fluency and confrontational naming were associated with various aspects of dual-task stride time variability and dual-task stride regularity, whereas vocabulary showed the strongest associations with dual-tasking pace (see Table 5). Previous studies have consistently found language to be associated with gait characteristics that load on the pace domain (Holtzer et al., 2006, 2012; Duff et al., 2007; Morris et al., 2016). Language, specifically relating to vocabulary tests, represent crystallized knowledge and generally remain stable or improve with age (Habeck et al., 2016). Vocabulary is generally used as one of the proxy measures of cognitive reserve (Stern, 2012). The strong association between vocabulary and gait may be linked to cognitive reserve. However, this requires further empirical testing. It is important to note that the nature of the dual task used in the present study was language-based and may, therefore, be driving the associations between language and mobility. Perhaps, if a non-language based task was selected (such as a visual scanning, digit span, auditory scanning task or serial subtractions) the results may change. Nevertheless, the language-based task often chosen for the dual-task condition across studies is intended to mimic real-life, wherein individuals frequently talk while walking.

For processing speed, both paper-and-pencil and computerized versions of the digit symbol tasks were associated with stride time variability and gait asymmetry under dual-task conditions (see Table 5), whereas a processing speed task involving components of visual scanning and mental switching was associated with turn duration during the TUG test. Previous studies have also found processing speed to be associated with various aspects of gait including pace, postural control and rhythm (Morris et al., 2016). The association between processing speed and various gait characteristics is not surprising given the greater reliance of both these functions on shared motor pathways. In support of this finding, recent papers using functional neuroimaging techniques have found that walking under single- and dual-task conditions was similarly associated with sensorimotor, vestibular and visual networks, with greater connectivity in the left fronto-parietal network including the supplementary motor areas during the dual-task condition (Yuan et al., 2015).

Interestingly, the majority of the cognitive-mobility associations were related to dual-task conditions and to turning duration. This finding suggests that in healthy individuals the association between cognition and gait is dependent on the complexity of the task. Perhaps in the simple, single-task condition, gait is relatively more automatic and less cognitively demanding, at least among healthy adults. However, with a cognitive load (e.g., talking while walking, during turns), both cognitive and mobility processes become impaired (Odonkor et al., 2013). One relatively straightforward explanation of the cognitive-motor dual-tasking effect is the capacity sharing theory. This theory proposes that simultaneously performing two attention-demanding tasks will cause the performance of one or both of the tasks to suffer due to limited information processing (Li and Lindenberger, 2002; Li et al., 2005, 2018). A second explanation, as detailed in a recent review paper (Li et al., 2018), proposes an explanation related to the Principle of Neural Overlap – viz.-a-viz., during the dual-task condition, both the motor and cognitive components of the task may engage shared neural pathways, thus leading to overall reduced costs. A third related explanation comes from functional neuroimaging studies which have found that increased bilateral activation of the prefrontal regions was associated with lowered performance during the dual-task condition, while increased left prefrontal region activation was linked with better performance (Li et al., 2018). Complementing these findings, a recent structural neuroimaging study (Tripathi et al., 2019) found that single and dual-task conditions were differentially involved with brain networks (gait during the single task condition was associated with supplementary motor area, precuneus cortex, and the middle frontal gyrus, whereas dual-task gait with medial prefrontal, cingulate, and thalamic regions).

In summary, executive function, perceptual speed, and language were associated with specific facets of mobility, especially during dual-tasking. These associations suggest that different combinations of physiological/cognitive systems are operating to regulate specific mobility domains. For example, different connectivity for specific aspects of mobility such as gait speed and gait variability have been identified (Lo et al., 2017; Tripathi et al., 2019). The current findings lend itself to further exploration of different brain pathways. Specifically, the overlapping association of stride regularity during a dual task with language and processing speed suggests the involvement of a different cortical circuity as compared to that involving the association of turn duration with executive function and processing speed. Research with specific biomarkers or clinical conditions affecting different cortical systems could shed light on whether the motor versus cognitive pathways are parallel, overlapping/intersecting, or causal. For example, it is known that slow walking speed is one of the features of frailty (Fried et al., 2001). The combination of specific gait and cognitive measures may aid in the early identification of the multisystem dysregulation associated with frailty. Another approach could be to compare the brain regions along with the cognitive-mobility phenotypes in known patient groups (e.g., Parkinson’s disease versus progressive supranuclear palsy) to better understand the role of shared substrates in these clinical syndromes (Hong et al., 2015). Additionally, a better understanding of the possible mediators associated with cognitive reserve and brain reserve on these cognitive-motor correlations may shed light on possible interventions that can impact these relations. One can hypothesize, for example, that brain reserve may contribute to inter-individual variation in cognition, and these may differentially affect the cognitive-motor associations. Similarly, the cognitive-motor associations might be influenced by the differential ability to cope with brain changes, which would implicate cognitive reserve. Future work that extends the present findings to examine the roles of cognitive and brain reserve will be informative.

## Conclusion

In the current study, we investigated a complex matrix of cognitive-mobility relationships in healthy adults in order to identify unique patterns. Our results, based on rather strict statistical criteria to avoid false positive findings, extend previous findings highlighting the role of executive functioning, language, and processing speed in dual-task walking conditions and for both turns and transitions that are proposed to be putatively independent mobility domains. Given the converging line of evidence, we have now increased the confidence in the findings of cognitive-mobility associations among healthy adults. The inclusion of other cognitive domains such as visuospatial functioning in future studies can help further elucidate these patterns. It will also be interesting to contrast the results of the present study with those using a global composite measure of cognition.

This present analyses was based on a cross-sectional study. Although gait and cognition are apparently interdependent, the study design makes it challenging to infer causality. More research across various clinical conditions and with longitudinal and experimental study designs will help to shed light on this issue. Another interesting line of work can also seek to examine the neuroimaging correlates and cortical control of these cognitive-mobility relationships. Additionally, the influence of possible genetic and environmental factors on cognition, mobility, and their relationships should be carefully investigated in follow-up investigations. Future studies may consider using a more stratified approach to investigating the cognition-mobility relationships by examining subdomains of cognition, such as contextual versus non-contextual verbal memory, verbal versus non-verbal memory, and immediate versus delayed memory. Nevertheless, the current study provides insight into specific features of motor-cognitive interactions and their impact per domain in aging and sets the stage for these future studies.

The implications of our finding are especially important for older adults. In aging and disease, as both cognitive functions and mobility decline, these two factors often interact with each other and increase the risk of adverse events and conditions (e.g., falls, frailty, disability, cognitive decline). Therefore, it becomes imperative to provide specific targeted interventions to improve cognitive, motor function and their inter-relationships that can subsequently result in improved functional mobility and cognition. Ultimately, the nature of such findings can improve the detection of early mobility and cognitive changes in older adults, and thereby enhance the development of novel therapies for those with clinical impairments.

## Data Availability

The datasets generated for this study are available on request to the corresponding author.

## Ethics Statement

The Internal Review Board of the College of Physicians and Surgeons of Columbia University approved this study. Prior to the testing session, written informed consent was obtained from all participants and compensation was provided at the end of the study.

## Author Contributions

PS, IM, ZA, JH, AM, and YS conceived the concept and rationale for the study. ZA collected the data. PS, YS, IM, and TK analyzed the data. PS, IM, JH, AM, and YS interpreted the results and drafted the manuscript. All authors participated in the approval of the final version of the manuscript and take responsibility for the content and interpretation of this manuscript.

## Conflict of Interest Statement

The authors declare that the research was conducted in the absence of any commercial or financial relationships that could be construed as a potential conflict of interest.

## Abbreviations

DT: dual task
MCI: mild cognitive impairment
TUG: timed up and go.
