# Supplementary material for: Differential Associations Between Distinct Components of Cognitive Function and Mobility: Implications for Understanding Aging, Turning and Dual-Task Walking
Source: Front Aging Neurosci. 2019 Jul 2;11:166. doi: 10.3389/fnagi.2019.00166 (PMC6614511; doi:10.3389/fnagi.2019.00166)
Supplement: Supplementary file 1 [file Table_1.DOCX]

| **Supplementary Table 1: List of full transformations applied to the cognitive and mobility variables.** | | | |
| --- | --- | --- | --- |
| **Variable Name** | **Original Yule Index Value** | **New Yule Index Value** | **Transformation Used** |
| AMNART | 0.44 | 0.19 | $SQRT(x)$ |
| Walking Amplitude | 0.20 | -0.03 | $SQRT(x)$ |
| Category Fluency (total correct) | 0.09 | 0.02 | $SQRT(x)$ |
| Antonyms (Proportion On Time Correct) | 0.07 | 0.07 | $None$ |
| Sit to stand jerk anterior-posterior | 0.81 | 0.06 | $SQRT(x)$ |
| Sit to stand jerk anterior-posterior | 0.35 | -0.05 | $log(x)$ |
| Sit to stand range anterior-posterior | 0.78 | -0.13 | $\frac{1}{SQRT\left( x \right)}$ |
| Sit to stand range anterior-posterior | 0.09 | -0.03 | $SQRT(x)$ |
| Timed up go anterior posterior duration | 0.02 | 0.02 | $None$ |
| WAIS-3 BD (total correct) | 0.05 | 0.05 | $None$ |
| Phonemic Fluency (mean correct) | 0.02 | 0.02 | $None$ |
| CPT Correct Ratio | 0.58 | -0.58 | $None$ |
| Digit Symbol (median correct RT) | 0.01 | -0.01 | $None$ |
| Flanker (adjusted composite score) | 0.35 | -0.35 | $None$ |
| GP-D, total time (in secs) | 0.35 | 0.02 | $\frac{1}{x}$ |
| GP-NP, total time (in secs) | 0.44 | 0.05 | $\frac{1}{x^{2}}$ |
| Dual task stride average | 0.16 | 0.01 | $\frac{1}{x}$ |
| Single task stride average | 0.10 | -0.01 | $\frac{1}{SQRT\left( x \right)}$ |
| Dual task stride time variability | 0.48 | 0.06 | $log(x)$ |
| Single task stride time variability | 0.32 | 0.04 | $log(x)$ |
| Letter Comparison (median correct RT) | 0.13 | 0.01 | $\frac{1}{SQRT\left( x \right)}$ |
| Letter Set (Proportion On Time Correct) | 0.19 | 0.19 | $None$ |
| Logical Memory (Proportion On Time Correct) | 0.36 | 0.36 | $None$ |
| Matrix Reasoning (Proportion On Time Correct) | 0.18 | 0.18 | $None$ |
| Paired Associates (Proportion On Time Correct) | 0.07 | 0.07 | $None$ |
| Paper Folding (Proportion On Time Correct) | 0.12 | 0.12 | $None$ |
| Pattern Comparison (median correct RT) | 0.09 | 0.00 | $log(x+1/6)$ |
| Picture Naming (Proportion On Time Correct) | 0.15 | 0.15 | $None$ |
| Sit to stand pitch amplitude | 0.06 | 0.06 | $None$ |
| Sit to stand pitch amplitude | 0.18 | -0.18 | $None$ |
| Sit to stand pitch amplitude | 0.24 | 0.06 | $SQRT(x)$ |
| Sit to stand pitch amplitude | 0.01 | -0.01 | $None$ |
| Set-Shifting, shift trials (ms) | 0.19 | -0.19 | $None$ |
| SRT Delayed Recall | 0.19 | -0.19 | $None$ |
| SRT Last Trial (total correct) | 0.34 | -0.34 | $None$ |
| Dual task step symmetry | 0.06 | -0.06 | $None$ |
| Single task step symmetry | 0.21 | 0.00 | $x^{2}$ |
| Dual task step regularity | 0.00 | 0.00 | $None$ |
| Single task step regularity | 0.18 | -0.02 | $x^{2}$ |
| Dual task stride length | 0.32 | 0.02 | $\frac{1}{x^{2}}$ |
| Single task stride length | 0.39 | -0.09 | $\frac{1}{x^{2}}$ |
| Dual task stride regularity | 0.24 | -0.02 | $x^{2}$ |
| Single task stride regularity | 0.16 | -0.06 | $x^{2}$ |
| Synonyms (Proportion On Time Correct) | 0.21 | 0.21 | $None$ |
| Timed up go duration | 0.17 | -0.03 | $log(x)$ |
| TMT-A (time in secs) | 0.23 | -0.02 | $log(x)$ |
| TMTB (time in secs) | 0.41 | -0.03 | $\frac{1}{SQRT\left( x \right)}$ |
| Single task speed | 0.58 | -0.29 | $\frac{1}{x^{2}}$ |
| WAIS-3 MR (total correct) | 0.15 | 0.05 | $x^{2}$ |
| WAIS-R DS (total correct) | 0.07 | -0.01 | $SQRT(x)$ |
| WAIS-R Vocabulary | 0.37 | -0.37 | $None$ |
| WCST Perseverative Errors (total score) | 0.67 | -0.07 | $\frac{1}{SQRT\left( x \right)}$ |
| Word Order (Proportion On Time Correct) | 0.19 | 0.19 | $None$ |
| WTAR (total correct) | 0.42 | -0.29 | $x^{2}$ |
| Turn yaw amplitude | 0.17 | -0.17 | $None$ |
| Turn yaw amplitude | 0.38 | -0.38 | $None$ |
| Turn yaw duration | 0.07 | -0.03 | $SQRT(x)$ |
| Turn yaw duration | 0.18 | 0.04 | $\frac{1}{SQRT\left( x \right)}$ |

**Supplementary Table 2: List of Outliers Removed from the dataset**

| **Variable** | **Outlier.ID** | **Original value** |
| --- | --- | --- |
| Logical Memory (Proportion On Time Correct) | 4909 | 0.067 |
| WAIS-R Vocabulary | 4883 | 17 |
| CPT Correct Ratio | 4245 | 76 |
| Flanker (adjusted composite score) | 4635 | 5.636 |
| Flanker (adjusted composite score) | 4508 | 5.945 |
| Dual task stride length | 4635 | 0.759 |
| Dual task time stride variability | 4252 | 14.755 |
| Single task step symmetry | 4378 | 1.393 |
| Dual task step symmetry | 4335 | 2.255 |
| Sit to stand range anterior-posterior | 4545 | 0.088 |
| Sit to stand range anterior-posterior | 4930 | 1.999 |
| Sit to stand pitch amplitude | 4645 | 99.766 |
| Sit to stand pitch amplitude | 5002 | 91.691 |
| Sit to stand pitch amplitude | 5002 | -85.902 |
| Sit to stand pitch amplitude | 4645 | 88.714 |
| Sit to stand pitch amplitude | 4545 | 1.633 |
| Turn yaw amplitude | 4545 | 0.128 |
| Turn yaw duration | 4394 | 4.07 |
